# Self-Directed Telehealth Parent-Mediated Intervention for Children With Autism Spectrum Disorder: Examination of the Potential Reach and Utilization in Community Settings

**DOI:** 10.2196/jmir.7484

**Published:** 2017-07-12

**Authors:** Brooke Ingersoll, Katherine Shannon, Natalie Berger, Katherine Pickard, Bree Holtz

**Affiliations:** ^1^ Michigan State University Department of Psychology East Lansing, MI United States; ^2^ Michigan State University Department of Communication and Public Relations East Lansing, MI United States

**Keywords:** autism, parenting education, telemedicine

## Abstract

**Background:**

There is a significant need for strategies to increase access to evidence-based interventions for children with autism spectrum disorder (ASD). One novel approach is to train parents to use evidence-based interventions for their child with ASD via telehealth. Pilot work examining the efficacy of one such program, ImPACT Online, demonstrated a high rate of parent program engagement, low attrition, and associated gains in parent learning and child social communication.

**Objective:**

The objective of this study was to conduct an open trial of ImPACT Online to better understand its dissemination potential.

**Methods:**

We examined the reach and representativeness of families who registered (n=36) compared to families who were referred (n=139) to the open trial for one referral site. We then compared the demographics of all families who enrolled in the open trial (n=112) to families who enrolled in one of two controlled trials of the same program (n=50). We also examined metrics of program engagement for the open and controlled trials, the relationship between program engagement and changes in parents’ intervention knowledge, and program evaluation for the participants in the open trial.

**Results:**

In total, 25.8% (36/139) of the parents who were given information about the program at their child’s diagnostic feedback session registered with the program. The parents who enrolled in the open (OT) and controlled trials (CT), respectively, were similar in gender (OT: 84.8% (95/112); CT: 88% (44/50), female), marital status (OT: 80.4% (90/112) ; CT: 69.6% (32/46), married), education (OT: 58.0% (65/112); CT: 54.0% (27/50), college degree or higher), and employment status (OT: 58.0% (65/112); CT: 65.3% (32/49), employed outside the home). The child participants were similar in terms of gender (OT: 83.0% (93/112); CT: 76.0% (38/50), male) and race and ethnicity (OT: 38.4% (43/112); CT: 24.0% (12/50), minority). However, the mean chronological age of the child participants in the open trial group was significantly higher (Mean=60.0 months) than in the controlled trial group (Mean=43.0 months), with *t*_160_=5.22, *P*<.001. Parents in the open trial engaged with the program at a significantly lower rate than the controlled trial, *F*_3,81_=21.14, *P*<.001. Program engagement was significantly associated with gains in parent intervention knowledge across both the groups, beta=.41, *t*=2.43, *P*=.02. Participants in the open access trial evaluated the program highly, but several barriers were noted.

**Conclusions:**

These data suggest that additional strategies may need to be developed to support families in using telehealth-based parent-mediated intervention in community settings.

## Introduction

Autism spectrum disorder (ASD) is a chronic and pervasive neurodevelopmental disorder characterized by deficits in social communication and the presence of restricted and repetitive behaviors [1]. Individuals with ASD often require intensive and comprehensive intervention across the life span [2]. There has been a dramatic increase in the number of individuals diagnosed with ASD over the last two decades, with prevalence rates reaching 1 in 88 [3]. However, there has not been corresponding growth in the availability of evidence-based services, contributing to high levels of unmet service needs for individuals with ASD and their families [4]. These issues highlight the need for systematic research focused on developing and improving strategies for dissemination and implementation of evidence-based ASD services.

Parent-mediated intervention is one cost-effective and ecologically valid way to increase access to evidence-based ASD intervention. Numerous studies have established that parents can be successfully taught to use evidence-based strategies to improve their child’s social-communicative functioning [5]. Additional benefits of parent-mediated intervention include increase in generalization and maintenance of child skill, a reduction in parent stress, and an increase in family leisure time [6,7]. Yet, there continue to be barriers involved with the dissemination of parent-mediated intervention, including a shortage of trained professionals, limited financial resources and transportation, lack of child care, geographic isolation, lengthy waitlists, and extensive time commitments [8]. Thus, it is essential to consider the adaptation of evidence-based, parent-mediated interventions for non-traditional service delivery methods [9]. Telehealth and technology-based applications have the potential to augment or even replace traditional service models to increase access to evidence-based services [10]. Self-directed telehealth programs can provide a cost-effective means for intervention to be accessed from anywhere at any time [10]. They can deliver highly standardized instruction with fidelity, while also supporting individualized learning [11]. The use of self-directed telehealth programs to provide instruction in evidence-based interventions has been explored across health-related disciplines, disorders, and treatment approaches with promising outcomes [12]. Taken together, these data suggest that telehealth applications may serve as a promising alternative service-delivery model to increase the reach of evidence-based ASD practices, including parent-mediated intervention [13].

Although there has been growing interest in using telehealth to deliver parent-mediated intervention for children with ASD, empirical evaluations of such programs are limited. Several recent studies have demonstrated the initial efficacy of evidence-based, parent-mediated autism interventions when delivered by computer or over the internet, with or without therapist assistance [14-17]. One such program, ImPACT Online, is an interactive website that teaches parents to promote their child’s social communication within the context of play and daily routines [14,18,19]. The content was modified from Project ImPACT, an evidence-based social communication curriculum for young children with ASD [20]. ImPACT Online can be delivered as a self-directed intervention or with therapist assistance via videoconferencing. A recent pilot randomized controlled efficacy trial comparing the two formats demonstrated positive effects on parent learning and child social communication skills for both groups, although there was an added benefit of therapist assistance on some parent and child outcomes [14,18]. Although engagement with the program website was significantly higher among parents in the therapist-assisted group, parents in the self-directed group engaged with the program at a relatively high rate, with 69% completing the program [19]. There was also a significant positive relationship between program completion and improvements in parent intervention knowledge and fidelity, independent of therapist assistance, suggesting that the use of the self-directed website contributed to parent learning.

Self-directed telehealth interventions do not require a trained professional and can typically be administered at a much reduced cost. Thus, they have a strong dissemination potential [21] and may provide an effective method for increasing access to intervention for underserved families of children with ASD. At the same time, much is unknown regarding the potential impact such interventions may have should they prove efficacious in larger trials. For example, it is currently unclear whether families who participate in controlled trials of telehealth parent-mediated interventions are representative of families who would use these interventions in the community. In addition, little is known about the reach of such programs in the community or the representativeness of the community families who choose to enroll. Research on self-directed telehealth programs for other conditions suggests that they may have a more limited reach than anticipated. For example, only 5% of health plan participants recruited by personal letter enrolled in a treatment trial of a Web-based weight loss program [22]. Further, those who enrolled in a Web-based weight loss program through their workplace were more likely to have higher household incomes, education, and health literacy than those who did not enroll, indicating disparities in access [23].

In addition, it is not clear whether the high rates of program engagement observed in our pilot trial of ImPACT Online would be observed in participants who openly enroll in the program. Attrition rates during open trials of self-directed telehealth interventions are often much higher than those found in controlled trials of the same intervention, in which potential participants are closely screened, monitored, and “pushed” to continue engaging with the program by the research staff [24]. For example, Christensen et al [24] found that less than 1% of individuals who enrolled in an open trial of MoodGym, a self-directed CBT-based telehealth program for depression, completed all five modules, compared with nearly 23% in their controlled trial; this represents a 450-fold increase in attrition among community participants.

Highly efficacious programs that have a limited reach or low quality implementation are not likely to have a significant impact on public health [25]. Therefore, it is important to evaluate these factors, alongside more traditional evaluations of program efficacy, to understand the potential impact that telehealth-based, parent-mediated intervention can have on access to care for underserved families of children with ASD [25]. This information will be critical to understanding the likelihood that this type of intervention will be able to reach the families for whom it is designed as well as identifying the required program modifications and supports necessary to facilitate its use and ultimate impact.

This study conducted an open trial of ImPACT Online to better understand the dissemination potential of a self-directed, telehealth-based, parent-mediated intervention for families of children with ASD. Specifically, we examined (1) the reach and representativeness of a subset of families who enrolled in the open trial, (2) the demographics of families who enrolled in the open trial compared to families who enrolled in one of our two controlled trials of the same program, 3) metrics of program engagement for the open and controlled trials, (4) the relationship between program engagement and changes in parents’ intervention knowledge, and (5) program evaluation for the open trial.

## Methods

### Participants

Participants in the open trial group (n=112) were parents of a child with ASD between the ages of 27 and 152 months who self-enrolled through the ImPACT Online website. To be included in the analyses, the participant had to identify as the child’s mother, father, or step-parent, live in the United States, be proficient in English, and complete the intake questionnaires. In addition, the child had to score above the cut-off on the Modified Checklist for Autism in Toddlers (M-CHAT; under 36 months) [26] or the Social Communication Questionnaire (SCQ; 36 months or older) [27]. An additional 101 registrants were excluded because they or their child did not meet one or more of these inclusion criteria.

Participants in the controlled trial group (n=50) were parents of a child with ASD between the ages of 19 and 73 months who had enrolled in the pilot (n=27) or full-scale efficacy trial (n=23, recruitment ongoing) of ImPACT Online. To participate, the participant had to identify as the child’s mother, father, or step-parent, live within 3 hours of the research lab, and be proficient in English. Inclusion criteria for the child included being between the ages of 18 and 95 months at intake, meeting criteria for ASD on the Autism Diagnostic Observation Schedule-2nd Edition (ADOS-2) [28], and having no known medical cause of ASD. Table 1 presents participant characteristics by group.

**Table 1:** Participant demographic information.

Characteristics	Group			
	Open trial	Controlled trial	Test statistics	*P* value
**Parent characteristics**				
Gender: female, n (%)	95 (84.8%)	44 (88.0%)	χ^2^_1_ (N=162)=0.3	.59
Education level: college degree, n (%)	65 (58.0%)	27 (54.0%)	χ^2^_1_ (N=162)=0.2	.63
Marital status: married, n (%)	90 (80.4%)	32 (69.6%)	χ^2^_1_ (N=158)=2.2	.14
Employment status: employed, n (%)	65 (58.0%)	32 (65.3%)	χ^2^_1_ (N=161)=0.8	.39
Internet literacy, mean (SD)	36.7 (3.5)	37.6 (3.9)	*t*_156_=-1.49	.14
**Child characteristics**				
Gender: male, n (%)	93 (83.0%)	38 (76.0%)	χ^2^_1_ (N=162)=1.1	.29
Race/Ethnicity: minority, n (%)	43 (38.4%)	12 (24.0%)	χ^2^_1_ (N=162)=3.2	.07
Chronological age in months, mean (SD)	60.0 (21.1)	43.0 (13.7)	*t*_160_=5.22	<.001
Mean intervention in hours / week (SD)	6.9 (9.2)	10.7 (10.1)	*t*_97_=-1.95	.05

### Study Procedures

#### Open Trial Group

Information about the open trial was disseminated via flyers given to families by professionals at community organizations serving children with ASD, websites providing information about ASD, and an Internet search. Community organizations who expressed interest were given recruitment materials with a unique site code which participants entered at the time of program registration to track referrals. Recruitment materials provided a link to the ImPACT Online website. The website described the content of the program, system requirements, research requirements, and allowed visitors to view a brief video demonstration of the program. Participants had to indicate their consent to participate in research before registering. After registration, participants were asked to complete several short pre-treatment questionnaires and were then given access to the program. 6 months after registration, participants were sent an email asking them to complete the post-treatment questionnaires. Non-responders were sent a follow-up email one week, and two weeks later, until a total of three emails were sent. Participants received a US $25 gift certificate for the completion of the post-treatment questionnaires. Throughout their participation, participants in the open trial received no individual contact with research staff.

#### Controlled Trial Group

Information about the pilot and subsequent full-scale efficacy trials was disseminated via community providers to families within 3 hours of the research lab. Interested parents were directed to contact the research lab to learn more about the specific study. Consent and intake assessments were conducted in person at the research lab and in the family’s home. All participants were provided a computer and high speed internet, if needed.

After intake assessments, pilot study participants were randomly assigned to a self-directed (n=13) or therapist-assisted group (n=14). Participants in the full-scale study were randomly assigned to a self-directed (n=6), therapist-assisted (n=8), or informational control group (n=9). Participants in the self-directed groups for both studies received periodic individual phone and email contact from project staff regarding research-related tasks throughout their participation, but they received no support in learning the intervention. Participants in the therapist-assisted groups for both studies received access to the ImPACT Online website in addition to twice-weekly coaching via videoconferencing to help them learn the intervention. Participants in the informational control group received access to an informational website and monthly support calls. Participants completed post-treatment questionnaires and assessments after roughly 6 months, and again at a 3-month follow-up. Participants received a US $25 gift certificate for the completion of assessments at pre- and post-treatment, and follow-up.

### Intervention

ImPACT Online is a secure, password-protected, interactive Web application. The content was adapted from Project ImPACT [20], a naturalistic, developmental behavioral intervention (NDBI) [29] that teaches parents to promote their child’s social communication during play and daily routines. The instructional content was presented in 12, self-directed lessons, each of which took approximately 75 minutes to complete. Each lesson contained a narrated slideshow (average length=27.2 minutes), written manual, self-check questions, video-based exercises, homework plan, and reflection questions. Parents were advised to practice the intervention with their child for one week following each lesson. Parents could also access supplemental material outside of the lessons, including a video library that contained longer video examples of the intervention, a moderated forum, and additional program handouts and informational resources. Parents received automated weekly emails that provided tips for implementing the intervention techniques along with a link to the program to encourage program use.

### Measures

#### Program Reach

One community organization, a university-based diagnostic center, tracked the number of referrals made to the program (recruitment flyers distributed during the diagnostic feedback session). The organization also tracked the age of the child in months, the child’s gender, and the family’s zip code. Zip codes were used to derive a median household income using US Census data for the referral group. This information was then compared with the same data provided by families who enrolled in the program using the referral site’s unique access code to calculate the potential reach of the program and the representativeness of the participants.

#### Participant Demographics

Participants were asked to provide information on their gender, education level, employment status, marital status, and zip code. They were also asked to provide information on their child’s gender, age, diagnostic status, the number of hours per week, and the type of intervention their child received.

#### Internet Fluency

Participants completed a brief, modified version of the Computer-Email-Web Fluency Scale [30] at pre-treatment to assess their level of comfort using the computer and Internet. Items such as (1) how frequently do you conduct a search using an Internet search engine, and (2) how frequently do you send or receive email, were rated on a 5-point scale from never (1) to daily (5). Total scores on the measure could range from 8 to 42, with higher scores indicative of greater comfort with using the Internet.

#### Program Engagement

Program engagement was measured via ImPACT Online’s electronic tracking of user behavior. Four metrics of program engagement were calculated: (1) average number of logins to the site, (2) average number of minutes spent on the site across the intervention period, (3) percent of learning activities visited at least once across the 12 lessons (out of a possible 71), and (4) program completion defined by having visited 75% or more of the learning activities. We also examined the percent of participants completing each lesson to determine whether there was a drop-off in program engagement associated with a particular lesson. Completion was defined as having clicked on 75% or more of the learning activities for a lesson at least once.

#### Parent Intervention Knowledge

Participants completed the ImPACT Knowledge Quiz, a 20-item, multiple-choice quiz, that assesses comprehension of the key elements of ImPACT at intake and approximately 6 months later to measure changes in their intervention knowledge.

#### Program Evaluation

Participants in the open trial were asked to complete several measures to evaluate their experience with the program and barriers to program engagement 6 months after registering with the site. The Behavioral Intervention Rating Scale (BIRS) is a well-validated measure that asks individuals to endorse items that assess the acceptability of a treatment’s procedures and its perceived effectiveness on a 6-point scale, ranging from 1 (highly disagree) to 3 (neutral) to 6 (highly agree) [31]. The BIRS was modified to better reflect the goals of the current intervention (acquisition of social-communication skills). Cronbach alpha for the BIRS was .97. The Website Usability Scale is a 10-item questionnaire developed for this project [19]. Participants rate the ease of use of the website and the perceived helpfulness of each program component on a 6-point scale, with higher scores indicating greater usability. Cronbach alpha for the website usability scale was .96. The Barriers to Treatment Participation Scale is a 44-item, well-validated measure of common barriers to participation in child outpatient treatment [32] *.* Questions are answered on a scale of 1 (“Never a problem”) to 5 (“Very often a problem”), with higher scores indicative of a greater number of perceived barriers. Items were modified to reflect potential barriers associated with the use of a self-directed, telehealth-based parent training intervention (as opposed to clinician-led, clinic-based therapy), resulting in the removal of 17 items assessing therapist- and clinic-related barriers, and the addition of 5 items assessing technology-related barriers. The modified scale contained 32 items and had a Cronbach alpha of .87. Participants were also asked to respond to 3 open-ended questions: (1) Please indicate any benefits of this program, (2) Please indicate any limitations of this program, and (3) Please provide any recommendations for improving this program.

## Results

The data were examined for normality by inspecting the skewness coefficient. Number of logins and the amount of time spent on the site were both positively skewed; a square root transformation was used to normalize their distributions. Intervention knowledge at post-treatment was negatively skewed; an arcsine transformation was used to normalize intervention knowledge at pre and post-treatment. All other data were normally distributed.

We first examined the reach of ImPACT Online for one referral site by comparing the number of parents who registered for ImPACT Online with the number of parents who were provided a referral from a professional at the time of diagnosis at that site (n=139). A total of 36 parents (25.8%) who were given information about ImPACT Online at the time of their child’s diagnosis registered with the site. Child gender, age, and median household incomes per year were compared for parents who registered (participant sample) and the referral sample. No significant differences were found between the two groups regarding gender distribution (Referral sample: 73.8% male; Participant sample: 71.4% male), χ^2^_1_ (N *=* 161) =.1, *P*=.78, child age in months (Referral sample: mean=41.8, SD=12.5; Participant sample: mean=41.7, SD=11.6), *t*_34_=−.03, *P*=.98, or median income (Referral sample: mean=$48,379, SD=$13,685; Participant sample: mean=$52,528, SD=$16,730), *t*_35_=1.49, *P*=.15).

Next, we compared the demographics of families who enrolled in the open trial (OT; n=112) with the controlled trial studies (CT; n=50), regardless of whether they had completed the 6-month follow-up period or their group assignment in the lab-based trials. As shown in  Table 1, parent participants in both groups were primarily female (OT: 84.8% (95/112); CT: 88% (44/50)), married (OT: 80.4% (90/112); CT: 69.6% (32/46)), slightly over half in each group had a college degree or higher (OT: 58.0% (65/112); CT: 54.0% (27/50)), and were employed full or part time (OT: 58.0% (65/112); CT: 65.3% (32/49)). Scores on the CEWFS ranged from 25 to 42, with average scores toward the upper end of the scale for both groups (OT: mean=36.7, SD=3.5; CT: mean=37.6, SD=3.9). Chi-square analyses and independent sample *t* tests revealed no significant group differences on any of these parent demographic variables.

The majority of child participants in both groups were male (OT: 83.0% (93/112); CT: 76.0% (38/50)), and rates did not significantly differ between groups. The children’s mean chronological age was significantly higher in the open trial (mean=60.0, SD=21.1) than the controlled trial (mean=43.0, SD=13.7), *t*_160_=5.22, *P*<.001, likely due to the age restriction of the controlled trials. Children in the open trial were marginally more likely to be a racial or ethnic minority than the lab-based trials (OT: 38.4% (43/112); CT: 24.0% (12/50)), χ^2^_1_, (n=162) =3.2, *P*=.07, which could reflect the general demographics of the recruitment area for the controlled trials.

Participants in both groups reported that their children received a variety of different intervention services (eg, special education, ABA, speech therapy, occupational therapy, play groups), with intensities ranging from 0 to 41 hours per week. However, children in the open trial (mean=6.9, SD=9.2) received marginally fewer hours per week of intervention than children in the controlled trials (mean=10.7, SD=10.1), *t*_97_=−1.95, *P*=.05.

Next, we examined program engagement metrics for the subset of participants in the open trial who had completed the 6-month follow-up period (n=94). A number of participants who enrolled in the open trial did not access the program after completing the pre-treatment assessments. Thus, we only included data from participants who accessed the first lesson (n=68, 61% of sample). In our previous work, therapist assistance was shown to enhance parent engagement in the program [19]. Thus, in order for the data to be comparable across studies, only the participants in the controlled trials who were assigned to the self-directed group and had completed the follow-up were included (n=17).

We examined group differences on the first three metrics of program engagement using MANOVA (Multivariate analysis of variance). There was a significant effect of trial type on program engagement, *F*_3, 81_=21.14, *P*<.001; Wilk’s Λ=.53, ƞ=.47. As observed in Table 2, the participants in the open trial engaged significantly less with the program on all of these metrics than the controlled trials. In addition, only 12% of participants in the open trial completed the program compared with 88% of participants the controlled trials, which represents a significant group difference, χ^2^_1_ (N=85)=40.3, *P*<.001.

**Table 2:** Program engagement.

	Group^a^			
	Open trial	Controlled trial (self-directed)	Test statistic	*P* value
Number of logins, mean (SD)	9.9 (8.5)	29.8 (13.3)	*t*_83_=−6.95^b^	<.001
Amount of time on site, mean (SD)	188.1 (197.1)	701.3 (371.0)	*t*_83_=−7.09^b^	<.001
% Learning activities completed, mean (SD)	26.3 (27.2)	88.6 (26.0)	*t*_83_=−8.55	<.001
% Participants completing program	12%	88%	χ^2^_1_ (N=85)=40.3	<.001

^a^Raw values.

^b^analysis used transformed data.

**Figure 1:**
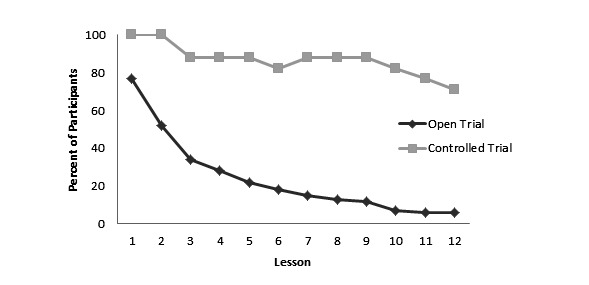
Percent of participants completing each lesson by group.

To better understand the rate of attrition, we examined the percent of participants who completed each lesson for the two groups. As observed in Figure 1, attrition in the open trial group occurred early in the program, with majority of participants discontinuing the program by the second lesson. In contrast, participants in the controlled trials maintained a high rate of engagement across the lessons.

We examined the degree to which program engagement was predictive of gains in parent intervention knowledge. We included those participants in the open trial (n=26, 38%) and the self-directed groups of controlled trials (n=16, 94%) who had completed the post-treatment questionnaire. For this analysis, we used a hierarchical linear regression, with parents’ intervention knowledge at the 6-month follow-up as the dependent variable. Parents’ pre-treatment intervention knowledge and trial type were entered in the first step. Program engagement, defined as percent of learning activities completed, was entered in the second step. After controlling for pre-treatment intervention knowledge and trial type, program engagement was a significant predictor of post-treatment intervention knowledge, beta=.41, *t*=2.43, *P*=.02, and explained additional variance in post-treatment intervention knowledge (*R2* change=.10, *F*=5.88, *P*=.02). This finding suggests that regardless of the trial type, participants who completed more of the program made greater improvements in their intervention knowledge from pre- to post-treatment.

Finally, we examined the program evaluation measures for the participants in the open trial (n=26). Participants reported the program as highly acceptability on the BIRS (mean=4.6, SD=1.0) and the website as highly usable (mean=4.9, SD=1.0). Parents also reported low levels of perceived barriers on the modified BTPS (mean=1.9, SD=.5). To understand the most common perceived barriers to using this program better, we examined the means for individual items on the modified BTPS. The three barrier items with the highest means (means ≥2.5) were: (1) “I did not have the time to practice (complete the homework)” (mean=3.0, SD=1.2); (2) “During the course of treatment, I experienced a lot of stress” (mean=2.9, SD=1.4); and (3) “Crises at home made it hard for me to complete the program” (mean=2.8, SD=1.3).

The open-ended responses were analyzed by compiling responses and identifying emergent themes using inductive content analysis. Two authors worked independently to develop codes based on the written responses. They then met to discuss initial codes and develop consensus regarding final codes. Any conflicts were discussed until consensus was reached. The most frequent themes identified in response to the program’s benefits, limitations, and recommendations are presented in Textbox 1.

**Open-ended response themes.**

Program Benefits

- Accessibility of the program
- Ease of learning the intervention
- Acceptability of intervention strategies
- Improvement in child social communication skills

Program Limitations

- Need for therapist support
- Lack of online community support
- Time requirements
- Stress in the home
- Technology barriers

Program Recommendations

- Teleconference support from coach
- Increase online community support
- Simplification of training components
- Make available on other platforms

## Discussion

### Principal Findings

In this study, we conducted an open trial of the self-directed format of ImPACT Online to understand its dissemination and implementation potential better. Our reach data indicate that only a quarter of parents who were provided information about the program at the time of their child’s diagnosis registered with the site. This finding is somewhat sobering given that parents in our pilot study reported that they would have liked to have received information about the program at the time of their child’s initial ASD diagnosis [18]. The fact that such a low percentage of families enrolled in the program may suggest that the diagnostic feedback session may not be the optimal time to provide a referral to this type of program. Parents of children with ASD consistently report that the diagnostic process is extremely stressful [33] and, while in retrospect, immediate access to parent-mediated intervention may sound attractive, at the time it may be perceived as too overwhelming. At the same time, the reach of telehealth interventions in general is poorly understood [34] and studies of eHealth interventions for other conditions, such as weight loss, have found much lower reach for their interventions, even among patients at high-risk for negative health outcomes [22]. So perhaps, the enrollment rate that we observed is, in fact, promising; however, without additional research in this area, it is difficult to know.

Our reach data were obtained from a specialty diagnostic clinic that provided a referral to the program at the time of diagnosis. It is unclear what the reach of this program would be in other communities or means of dissemination, such as advertisements, web-links, personal invitation via mail or email, or personal recommendation provided by an intervention provider. Different methods of dissemination may be expected to lead to different levels of enrollment as well as ongoing engagement with the program. Additional research that can examine differences in referral methods and the optimal times to refer parents to such programs would be helpful.

Although the data we were able to collect about families before they enrolled in the open trial were very limited, what we did collect suggested that those families who enrolled in the program were not significantly different from the referral sample in terms of median household income, child gender, or age. Although limited by sample size, this finding provides some indication that the families who enrolled in the open trial were fairly representative of the referral group. In addition, our demographic information suggests that, for the most part, participants who enrolled in our controlled trials were very similar to families who enrolled in the open trial. Although these data are, again, limited due to sample size constraints, they provide some confidence that the data being generated from our ongoing efficacy study may well generalize to community users. At the same time, there were few families with very low socioeconomic status in any of our samples, and thus, participation was not representative of the full range of families who could potentially benefit from this type of intervention. Therefore, future research should focus on increasing enrollment of parents from low socioeconomic status backgrounds into both open and controlled trials of telehealth-based, parent-mediated intervention.

Our program engagement data suggested much lower rates of engagement among our open trial participants than the participants in our controlled trials. This finding is not surprising given the previous research on telehealth-based interventions for other conditions. Participants in our controlled trials received a significant amount of personal contact with the research team throughout their participation, which may have affected their motivation to engage with the program. Although program engagement was much lower among participants in the open trial than the controlled trial, it was still higher than many open trials of similar interventions [35-37]. This may suggest a higher level of motivation among parents of children with ASD than individuals who are pursuing health promotion or mental health services for themselves.

It has been proposed that program completion may not be necessary to achieve significant improvements, in part because some individuals who discontinue eHealth interventions do so because they improve [38]. Inspection of the percent of participants completing each lesson suggests that there was an early decline in lesson completion among participants in the open trial. This is particularly problematic given that the first three lessons are focused on background information and the primary intervention content is not presented until lesson four, at which point fewer than 30% of participants completed the lesson. Thus, it appears unlikely that early attrition is due to improvements in this case. Although our data were limited due to a high percent of participants lost to follow-up (dropout attrition), we also found an association between the amount of program content accessed and increase in parent intervention knowledge, suggesting that that program engagement is an important factor in learning. This finding suggests a possible need to retool the content of the program to introduce intervention content earlier or for the use of additional strategies, particularly during the first few lessons, to boost engagement in real world contexts.

Our program evaluation data for the open trial participants suggested high levels of the treatment acceptability and website usability and low levels of perceived barriers. Our qualitative data complemented these findings, suggesting that parents identified a number of benefits of the program, including the accessibility of the program, and the ease of learning the intervention, the acceptability of the intervention strategies, and improvement in their child’s social communication skills. These themes have also been identified among participants in our controlled trial [18].

The most consistently reported barriers on the modified BTPS and open-ended questions were related to competing stressors at home and difficulty finding time to complete the program. These barriers might be expected to adversely impact parent participation in all forms child treatment, not just self-directed, telehealth-based parent-mediated intervention [39]. Our qualitative data also indicated a need for therapist support, simplification of some of the training components, and a desire for greater online community support. Therapist support was also identified as important theme among participants in our controlled trial [18]. However, simplification of training components and increased online community support were not reported in our controlled trial, suggesting that these factors may need to be considered in more detail, especially as self-directed programs are moved into community use.

### Future Research

The literature suggests a number of program design factors that may impact engagement in self-directed telehealth interventions, such as ImPACT Online [40]. First, the narrated slideshows, which were used to present the intervention content, were 27.2 minutes long, on an average. Guo and colleagues [41] have suggested that videos in an online learning environment should be no longer than 6 minutes. Although participants could stop and resume the videos at any point, breaking up the narrated slideshows may make the videos more engaging or easier to access in shorter increments. Likewise, ImPACT Online includes 12 lessons which are designed to be completed over the course of 3-6 months. This represents a significant time investment. It has been suggested that shorter, simpler interventions are better suited to online delivery than the more complex ones that require hours of online work [42]. Research identifying active ingredients of the ImPACT intervention could inform the development of a “leaner” intervention with fewer components which may be less prone to attrition.

Second, the Pew Research Center states that people are increasingly turning to their mobile phones to access the Internet [43]. Thus, making the website mobile compliant or developing an app component for those with a mobile phone would allow parents to access the lessons in any location. In addition, the use of personalized feedback or tailored messaging could be used to provide more individualized messages and content to the parent [44]. Another possibility would be to add some elements of gamification. Gamification uses elements of games in non-game contexts to support and engage individuals to complete tasks within a learning environment. These typically have been found to improve user engagement, learning, and ease of use [45]. There are myriad ways that this can be achieved, including giving points for users interacting with the content, achieving badges, and being included on a team. Past research has suggested that gamification can increase knowledge in health domains, self-efficacy, and motivation which can improve a myriad of health outcomes [46].

Finally, online communities may help improve peoples’ use of health content and interventions [47]. ImPACT Online included a moderated asynchronous forum; however, it was rarely used. It has been suggested that this type of online community may require a minimum threshold of activity to support ongoing site utilization [48]. Indeed, this was a reported limitation of the program. Currently, there are a multitude of online support groups available online, especially for parents of children with special needs [49]. Thus, it may be more practical to utilize currently available platforms. For instance, a closed and secret Facebook group is easier to develop, maintain and engage parents [50]. In addition to the parents being able to provide social support and exchange information, it is another way moderators can redirect parents back into the ImPACT Online website and content.

Future research that can evaluate which strategies are most likely to increase parent engagement in ImPACT Online and similar interventions is needed. In addition, as we did not collect data on other aspects of parent learning such as procedural fidelity, parent well-being, or child gains, future research should investigate how this program impacts important parent and child outcomes in real world settings.
